# Identifying Individuals by fNIRS-Based Brain Functional Network Fingerprints

**DOI:** 10.3389/fnins.2022.813293

**Published:** 2022-02-11

**Authors:** Haonan Ren, Shufeng Zhou, Limei Zhang, Feng Zhao, Lishan Qiao

**Affiliations:** ^1^School of Mathematics Science, Liaocheng University, Liaocheng, China; ^2^School of Computer Science and Technology, Shandong Technology and Business University, Yantai, China

**Keywords:** functional near-infrared spectroscopy, brain functional network (BFN), cross-task, individual identification, cross-view

## Abstract

Individual identification based on brain functional network (BFN) has attracted a lot of research interest in recent years, since it provides a novel biometric for identity authentication, as well as a feasible way of exploring the brain at an individual level. Previous studies have shown that an individual can be identified by its BFN fingerprint estimated from functional magnetic resonance imaging, electroencephalogram, or magnetoencephalography data. Functional near-infrared spectroscopy (fNIRS) is an emerging imaging technique that, by measuring the changes in blood oxygen concentration, can respond to cerebral activities; in this paper, we investigate whether fNIRS-based BFN could be used as a “fingerprint” to identify individuals. In particular, Pearson's correlation is first used to calculate BFN based on the preprocessed fNIRS signals, and then the nearest neighbor scheme is used to match the estimated BFNs between different individuals. Through the experiments on an open-access fNIRS dataset, we have two main findings: (1) under the cases of cross-task (i.e., resting, right-handed, left-handed finger tapping, and foot tapping), the BFN fingerprints generally work well for the individual identification, and, more interestingly, (2) the accuracy under cross-task is well above the accuracy under cross-view (i.e., oxyhemoglobin and de-oxyhemoglobin). These findings indicate that fNIRS-based BFN fingerprint is a potential biometric for identifying individual.

## 1. Introduction

Identifying individuals from a group is a significant task that has largely related to social security system and health care system (Ginther et al., 1992; Schmidt et al., 2005; Gershon et al., 2009). The mainstream identification characteristics, including face, fingerprint, and so on, are easily counterfeited, unstable in time, and involve privacy implications (Prabhakar et al., 2003; Jain et al., 2004, 2006). Advanced studies indicate that brain functional network (BFN) estimated by the temporal correlation between pairs of brain regions has the advantages of anti-imitation and stability in timing (Hilger et al., 2017, 2020; Wang et al., 2019b; Sastry et al., 2021). More importantly, the BFN-based “fingerprint” provides a potential way of exploring the brain at the individual level.

Up to now, several modalities of data have been utilized for constructing BFN fingerprints (Finn et al., 2015; Wang et al., 2019a, 2020; da Silva Castanheira et al., 2021; Sareen et al., 2021), including functional magnetic resonance (fMRI), electroencephalography (EEG), and magnetoencephalography (MEG). Among these modalities, fMRI was first used to estimate and identify BFN “fingerprint” by Finn et al. (2015). Their experimental result indicated that the fMRI-based BFN fingerprint can lead to a high identification accuracy, and individual functional connectivity is intrinsic and reliable. After Finn's work, Wang et al. (2019a) represented EEG signals as BFNs and used the deep intrinsic features of BFNs captured by the graph neural network for subject identification, showing that BFNs demonstrated more robust biometric traits than univariate features such as power spectral density functions and coefficients of auto-regressive stochastic models. Furthermore, da Silva Castanheira et al. (2021) generated functional connectivity fingerprints from MEG that measures the resting-state brain activity, and achieved a similar recognition rates to fMRI in the individual identification task.

As a complementary functional neuroimaging technique to fMRI and MEG, the emerging functional near-infrared spectroscopy (fNIRS) fNIRS has successfully explored the functional activation of shallow cerebral cortex during human behavior (Quaresima and Ferrari, 2019). The fNIRS simultaneously provides the concentration changes in de-oxyhemoglobin (Deoxy-Hb) and oxyhemoglobin (Oxy-Hb), and the latter delivers additional information with respect to the fMRI signal (Irani et al., 2007; Duan et al., 2012). Also, the insensitivity of fNIRS to movements and the portability of the device make it possible for long-term monitoring and repeated measurements of cortical activities possible in various scenarios, such as outdoor activity or resting state. More importantly, the relatively low-cost and non-invasive technology makes the fNIRS applicable among larger groups, including infants and children (Strangman et al., 2002). Based on these advantages, fNIRS is naturally suitable for the study of individual identification under motion stimulating condition.

In this study, we mainly investigate whether the bio-specific BFNs extracted from fNIRS data are discriminative enough to identify individuals. More specifically, we use an open-access fNIRS dataset (Bak et al., 2019) from 30 subjects with multiple tasks, including resting state (REST), right-handed tapping (RHT), left-handed tapping (LHT), and foot tapping (FT) in this study. Note that we regard the resting state as a special task. The BFN fingerprints corresponding to each task are first calculated by Pearson's correlation (PC). Then, based on the nearest neighbor scheme, we demonstrate that an individual-specific BFN fingerprint extracted from one task can be used to match those from another. The results show that BFN fingerprints estimated from different tasks are strongly intrinsically linked and that they are stable and reliable biometric features for individual identification. Additionally, since the BFNs from different views (i.e., Oxy-Hb and Deoxy- Hb) are involved, we can naturally design cross-view experiment to explore the possibility of individual identification. Furthermore, we believe that BFN fingerprinting has a potential in the brain exploration and patient identification for medical systems, which also presents a viable thinking for decoding the brain functional states at the individual level.

The rest of this paper is organized as follows. In Section 2, we introduce the fNIRS data preparation, BFN fingerprints estimation, and their identification. In Section 3, we report the identification accuracy across different tasks. In Section 4, we analyze the experimental results and point out some limitations of the involved scheme. Finally, we summarize this paper in Section 5.

## 2. Materials and Methods

In this section, we describe the data preparation (including acquisition and preprocessing), BFN estimation, and BFN-based fingerprint identification.

### 2.1. Data Preparation

#### 2.1.1. Data Acquisition

In this paper, an open-access dataset of fNIRS with three kinds of tasks (including RHT, LHT, and FT) is used to conduct individual identification experiments. In particular, 30 subjects (23.4 ± 2.5 years old) participated in the experiment. All of them declared that they have no psychiatric and neurological disorder that could affect the experimental results. The data are freely downloaded from https://figshare.com, and more details about the dataset can be found in Bak et al. (2019).

**Figure 1:**
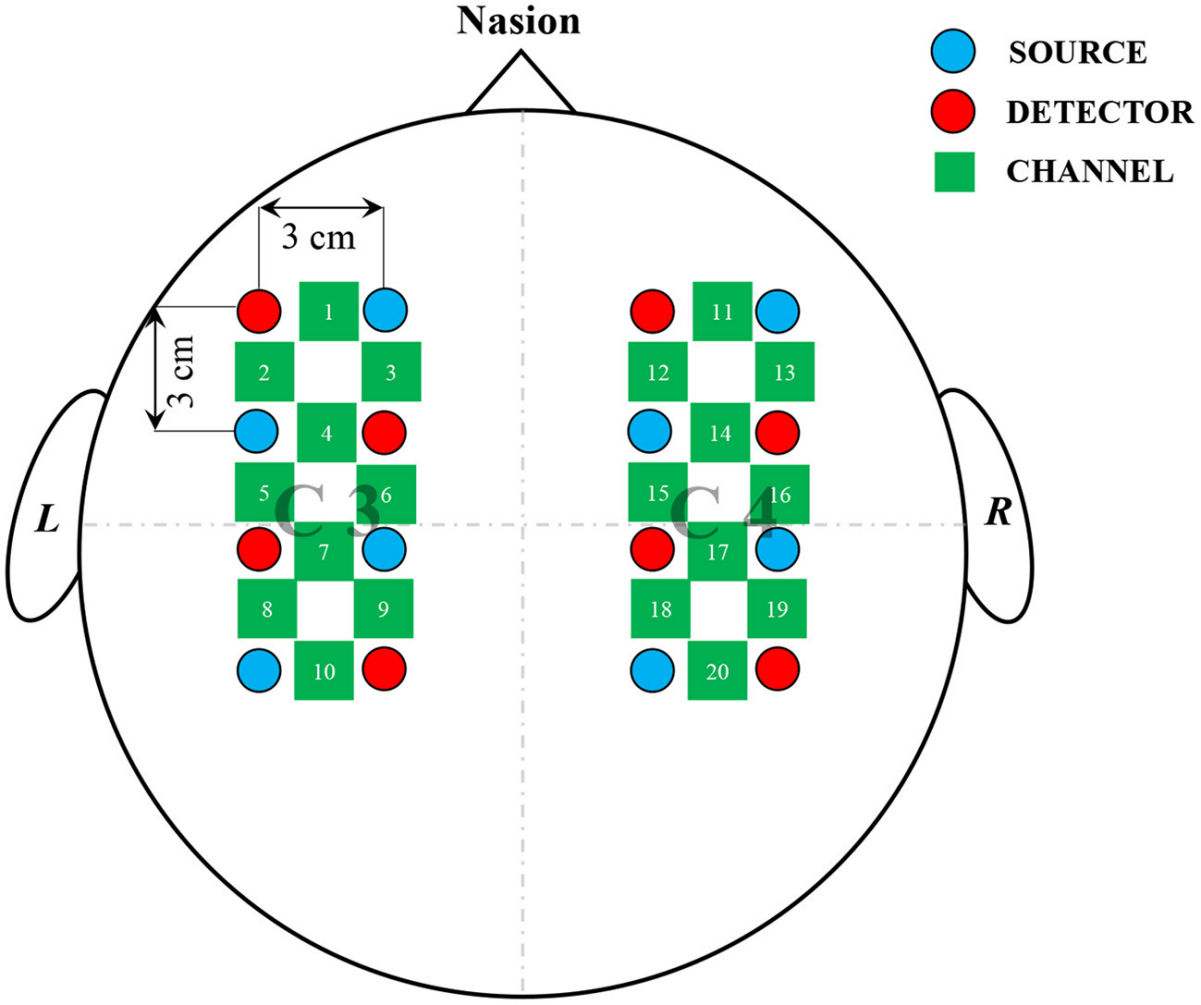
Arrangement of channels. Light sources and detectors were placed around C3, C4; red circles, blue circles, and green squares represented sources, detectors, and channels, respectively; total of eight sources and eight detectors, having separation of 3 cm between each source-detector pair, formed 20 channels to record cerebral activities.

Following the literature (Bak et al., 2019), the equipment used in the experiment was a three-wavelength continuous-time multi-channel fNIRS system (LIGHTNIRS, Shimadzu, Kyoto, Japan) consisting of eight light sources and eight detectors, which formed 20 channels to record changes in blood oxygen concentration. As shown in Figure 1, the light source and detector were located 3 cm apart and evenly distributed around C3 and C4 that represent the motor cortex (Georgopoulos, 1988; Gratton et al., 2006).

**Figure 2:**
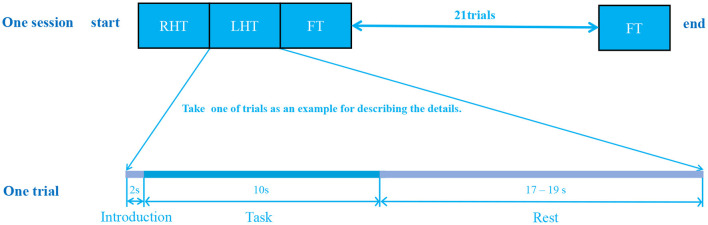
Paradigm for data acquisition. The data acquisition experiment was separated into three sessions, each session contained 25 trials, and each trial lasted about 30 seconds. As a result, for each subject, 75=25*3 trials were recorded with a complete experiment duration of about 2250 seconds = 75*30 seconds. Three tasks were involved, and each trial randomly selected one task to perform. A single trial consisted of three stages: 2-second introduction phase followed by 10-second task period and again followed by 17–19 seconds rest stage.

During the data collection, all subjects were informed to seat in front of a 27-inch monitor and executed random commands that appeared on the screen. The detection pipeline for a individual consisted of three sessions. Each session contained 25 trials, and each trial lasted an average of 30 s as shown in Figure 2. The 30-s trial consisted of three phases: the first 2 s were the introduction period, during which instructions appeared randomly on the screen; the next 10 s were the experiment period, during which the subjects need to make corresponding actions; and the last 17–19 s were the rest period, during which the brain returned to the resting state.

#### 2.1.2. Data Preprocessing

The original optical density information is collected using the multi-channel fNIRS system and need to be converted into the changes in blood oxygen concentration by modified Lambert–Beer law (Khan et al., 2020). Due to different motion artifacts (like heartbeat, respiration, and Mayer wave) and the interference from the instrument, the signals need to be further processed for the subsequent identification experiment (Delpy et al., 1988). First, a band-pass filtering is implemented by the third-order Butterworth filter with a cut-off frequency of 0.01–0.1 Hz to eliminate the physiological noise (approximately 0.1 Hz for Mayer wave, 0.25 Hz for respiration, and 1 Hz for a heartbeat). Then, the baseline correction is used to subtract the global signal (i.e., the average signals of all channels) from all signals (Nguyen et al., 2018; Zhang et al., 2021). All the preprocessing mentioned above is done through the BBCI toolkit (Blankertz et al., 2016).

Note that, in order to maintain data integrity, we do not abandon any channel signal (even with low signal-to-noise ratio) since they may contain some individual variability.

### 2.2. BFN Fingerprints Estimation and Identification

#### 2.2.1. BFN Estimation

**Figure 3:**
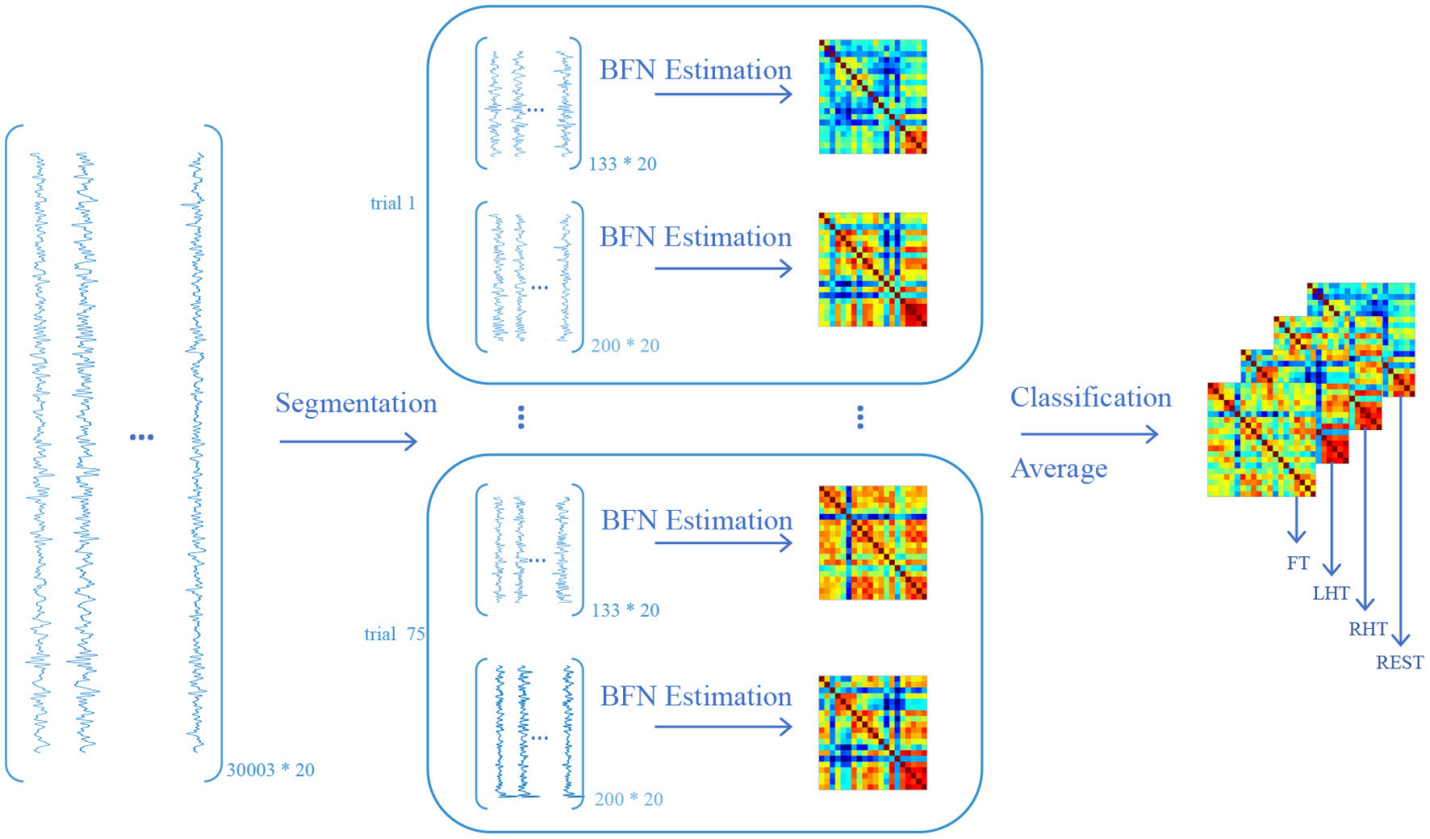
The pipeline of converting functional near-infrared spectroscopy (fNIRS) signals to brain functional networks (BFNs) (only take the first subject in the dataset as an example). The size of the original Oxy-Hb data is 30003*20, where the 30003 is the number of time points, and the 20 is the number of channels. By segmenting the original data according to the phase of task and resting state for each trial, we obtain 150 data matrices, half of which have a size of 133*20 under task state, and the remaining have a size of 200*20 under resting state. Note that, when conducting the segmentation operation, we truncate the time points that lie at task phase and the intermediate of the rest phase, respectively, for ensuring signal purity and eliminating the signal noise caused by task-resting switching. The data matrix is then used to estimate BFN fingerprint by PC. As a result, the subject obtains 150 BFNs, each of which has a size of 20*20. All BFNs are classified into four classes according to four tasks (i.e., RHT, LHT, FT, and REST). Each task class contains 25 BFNs and the resting class contains 75 BFNs. Since the size of each BFN is same, the subject would obtain a new BFN by averaging the element of the corresponding position of all BFNs within one class. Eventually each subject obtains 4 BFNs corresponding to 4 tasks.

After data preprocessing, we convert the signals into BFNs according to the pipeline shown in Figure 3. Since the involved subjects are treated in a similar way, we only take one subject under Oxy-Hb view in the dataset as an example to illustrate the conversion process. The first step is signal segmentation according to the trials. In consideration of the time point of one trial consisting of task and resting states, we segment task and resting data. As a result, we acquire 150 signal matrices, half of which correspond to task state and the remaining correspond to resting state. The second step is BFN estimation based on these signal matrices. In particular, the nodes of the BFN correspond to 20 channels, and the edge or edge weights are estimated as the PC between the time series (the columns of the signal matrix) associated with the channels. Finally, these estimated BFNs are divided into four groups according to the type of tasks (including RHT, LHT, FT, and REST), and the BFNs in each group are then averaged to generate a representative BFN for each task. Consequently, we acquire four BFNs for each subject, corresponding to four different tasks.

#### 2.2.2. BFN Identification

**Figure 4:**
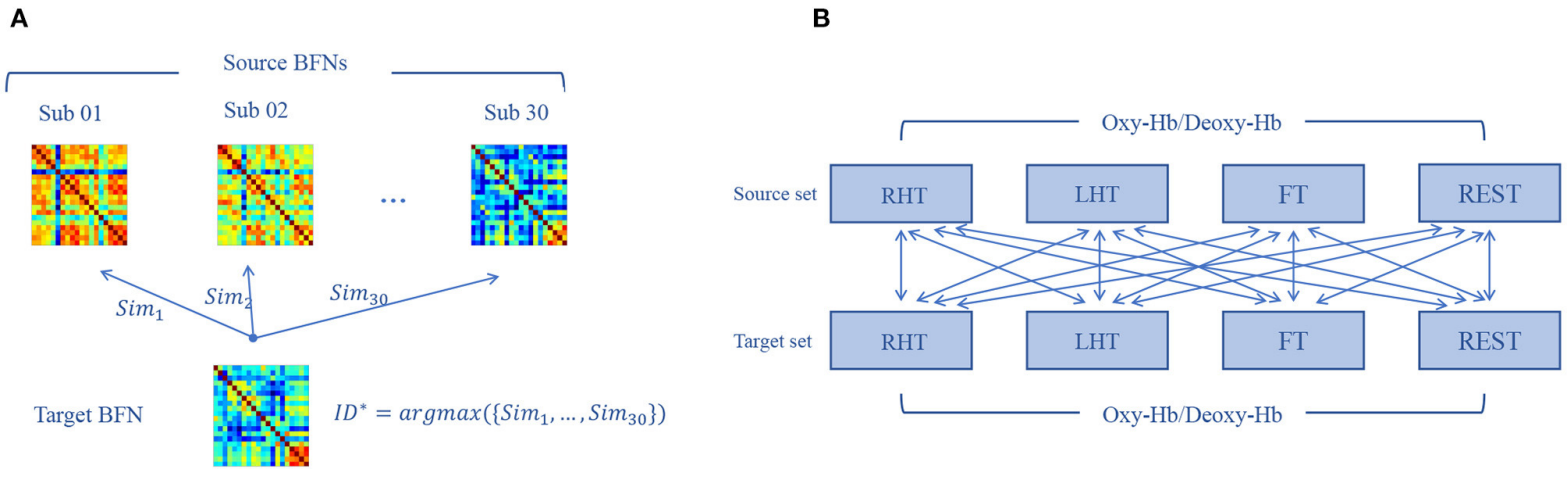
Individual identification procedure. **(A)** We calculate the similarity between a given target brain functional network (BFN) and all the source BFNs, respectively, and the label of the source BFN corresponding to the maximum similarity is the prediction label of the target matrix. **(B)** After data preprocessing, each subject obtains four BFNs corresponding to four tasks. By setting all BFNs from one state as source set and another state as target set for identification, we can get 16 possible combinations of the target-source separation.

Individual identification is performed across tasks based on the “source set” and “target set,” as shown in Figure 4A. In particular, given a target BFN $x_{t}^{*}$, we calculate its similarity to each BFN $x_{s}^{\left(i\right)}$ in the source set, denoted by $Sim\left(x_{t}^{*},x_{s}^{\left(i\right)}\right),i=1,2,\cdots \;,30$, where the similarity is defined as PC between two BFNs. Then, we use the nearest neighbor principle to predict the label ID^*^ of the target BFN as follows:


(1) $$\begin{array}{r} \begin{array}{cc} ID^{*}=arg\underset{i\in \left\{1,2,\cdots \;,30\right\}}{\max}\; & Sim\left(x_{t}^{*},x_{s}^{\left(i\right)}\right) \end{array} \end{array}$$

If the predicted label is equal to the actual label, the prediction score is counted as 1, and 0, otherwise.

After matching all pairs of BFNs in the target-source set, we can get the recognition accuracy as


(2) $$\begin{array}{r} ACC=\frac{the\;sum\;of\;prediction\;scores}{the\;total\;number\;of\;subjects} \end{array}$$

Since different tasks are involved in the dataset, we conduct cross-task individual identification experiment by setting all pairs of target-source mode, as shown in Figure 4B.

## 3. Results

**Figure 5:**
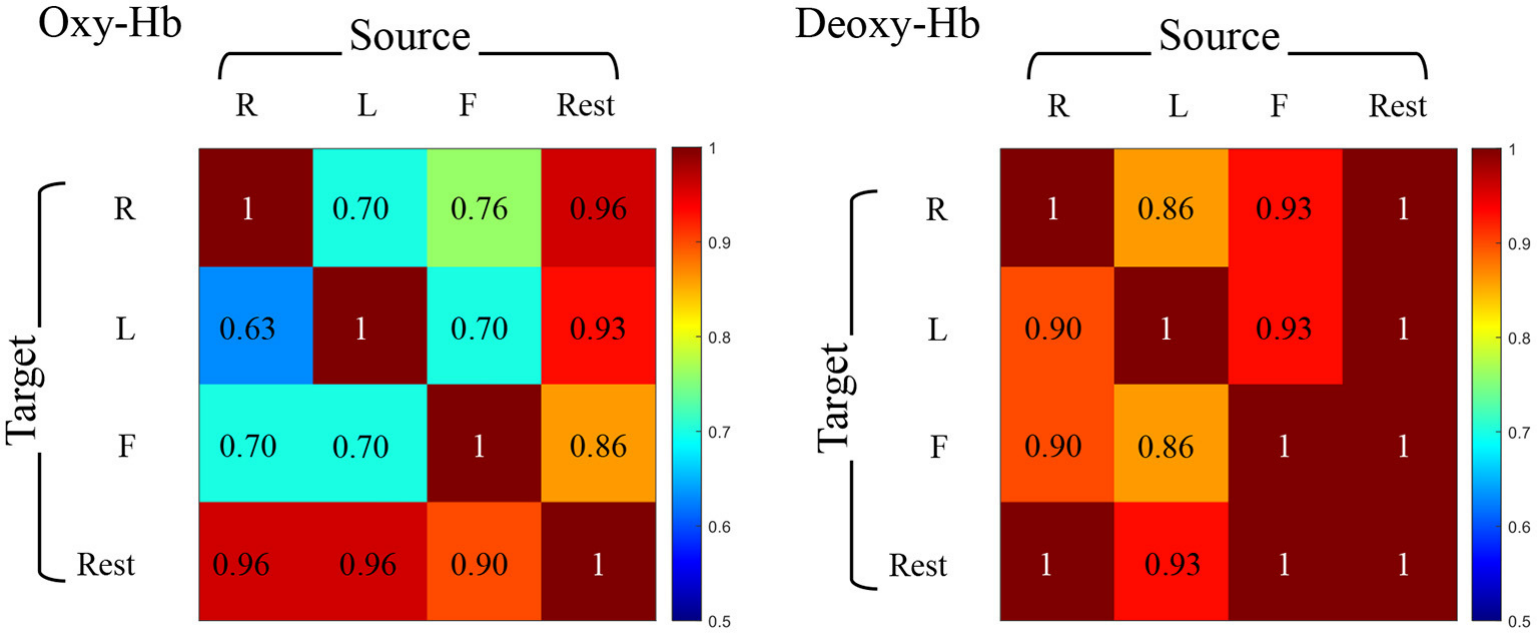
Accuracy of identification under cross-task condition. Individual identification accuracy for the source set from one task and the target set from another task. Each row represents the same target set and each column means the same source set. Oxy-Hb is on the left and Deoxy-Hb is on the right.

We eventually get the prediction accuracies within all pairs of target-source modes and the experimental results between the same views are shown in Figure 5. The accuracy varies from 20/30 (63%) to 30/30 (100%) in different modes (REST vs. RHT, REST vs. LHT, REST vs. FT, RHT vs. LHT, RHT vs. FT, LHT vs. FT for Oxy-Hb and Deoxy-Hb, respectively).

**Figure 6:**
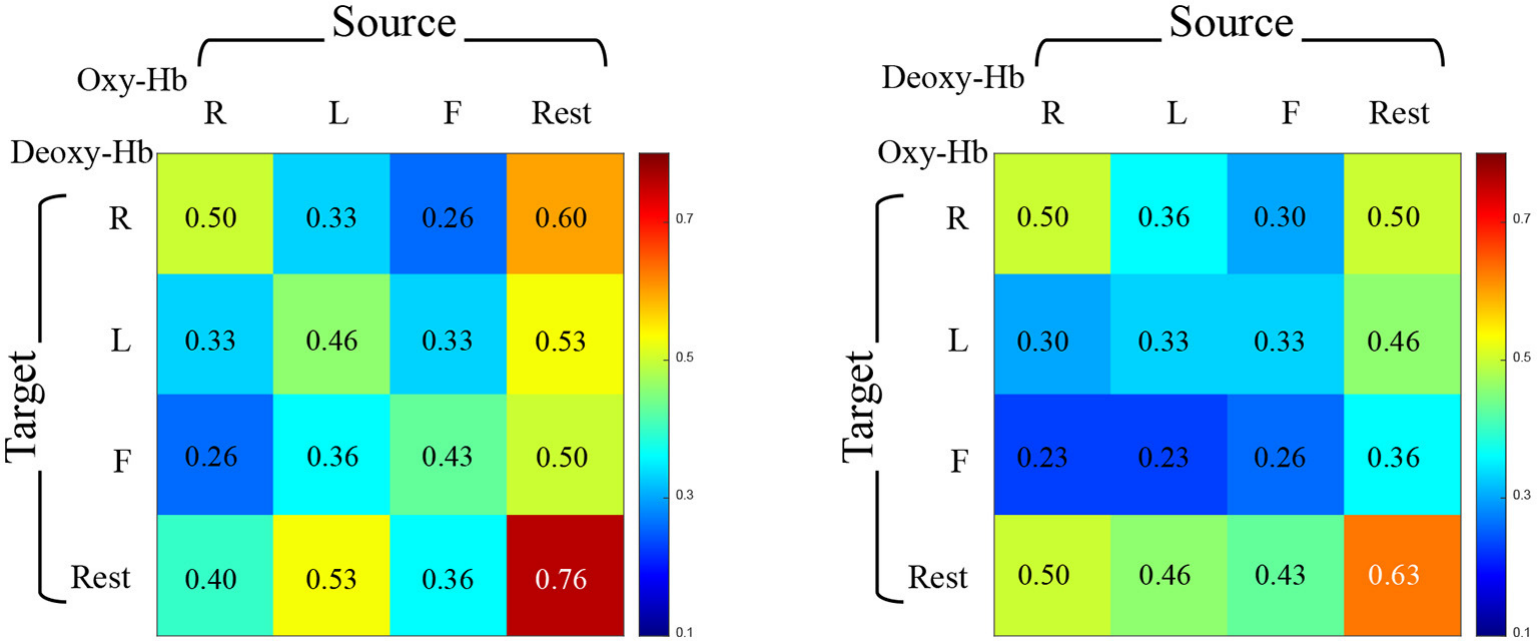
Accuracy of identification under cross-view condition. Individual identification accuracy between Oxy-Hb and Deoxy-Hb consists of four tasks. On the left, each row represents the target set of Deoxy-Hb, and each column represents the source set of Oxy-Hb. On the right side, it is the opposite.

Since the BFNs from different views are involved, we can naturally design cross-view experiment to place BFNs from Oxy-Hb and Deoxy-Hb into source/target sets, respectively. However, compared to the performance associated with the same views, the overall recognition accuracy under cross-view condition shown in Figure 6 changes significantly, ranging from 9/30 (23%) to 23/30 (76%). Meanwhile, we acquire the highest accuracy in REST–REST mode and the lowest accuracy in RHT–FT mode.

## 4. Discussion

### 4.1. Results Comparison

On the basis of experimental results, we have the following findings: (1) The identification accuracy under cross-task is 95.69% ± 0.29 for Deoxy-Hb and 86.00% ± 1.87 for Oxy-Hb, meaning that the identification performance based on Deoxy-Hb is better and more stable than Oxy-Hb in this experiment. Meanwhile, the identification accuracy under cross-view of using Deoxy-Hb as the source set to identify Oxy-Hb as the target set is higher than the opposite view setting. This finding encourages us to explore more potential on cross-view identification based on fNIRS data. (2) The accuracy under cross-task is well above the accuracy under cross-view. To explain this finding, we retrace the experimental process, and discover that the similarity between BFNs estimated under different tasks by one subject is much higher than the similarity between BFNs estimated under the same task state by different subjects. That is, the variability of BFNs is closely related to individual behavioral differences, but is more dependent on the inherent structure and function of the brain itself. (3) In most cases, the resting-state BFNs can be uniquely identified by a given BFN obtained from another task state. This phenomenon illustrates that the BFN in the rest phase is more suitable for individual identification than the BFNs in the task phase.

### 4.2. Limitations

However, there are some limitations that need to be considered. At the data level, in addition to the unavoidable small sample, all channels are located in motion cortex, which prevents us to estimate more representative BFN fingerprints based on the whole-brain changes in blood oxygen concentration. At the method level, we combine PC and the nearest neighbor scheme to conduct the identification experiment. In particular, PC is the simplest and wildly used method for estimating BFNs, but it captures the full correlation between pairs of channels and does not remove the confounding effect of other channels. According to previous studies (Hiwa et al., 2016; Guo et al., 2021; Sun et al., 2021; Xue et al., 2022), we can estimate more discriminative BFNs to identify individual. This is a direction for future research.

### 4.3. Additional Considerations

Note that, since the subjects' behaviors are constantly changing, BFNs are also vary dynamically and significantly within short periods of time (Hutchison et al., 2013). Hence, longer measurement time is one of the prerequisites for obtaining the discriminative and stable BFNs, and future work should focus on the correlation between the length of the signal and the discriminative nature of the BFN. In addition, we find that all signals of one subject in cross-task experiment are collected on the same day, and it is unclear to what extent does the interval between sessions affect the discriminative BFN fingerprints. Future work should focus on the stability or variability of BFN fingerprints over several months or years rather than days.

## 5. Conclusion

In this paper, we conduct individual identification experiments on fNIRS data under cross-task and cross-view conditions, respectively. The identification process includes the BFNs estimation and identification. In particular, we calculate PC between BFNs as similarity, and then evaluate the feasibility of subject recognition. The experimental results show that fNIRS-based BFN fingerprints have good bio-specificity and the properties of difficulty to imitation, which have the potential to serve as an alternative biometric feature for identifying individuals. However, this method, in this paper, only considers the similarity of BFNs estimated between different states or views, without mining the association between BFNs. Therefore, we plan to explore the consistency of BFNs based on fNIRS from the perspective of multi-task, even multi-view in the future.

## Data Availability Statement

The original contributions presented in the study are included in the article/supplementary material, further inquiries can be directed to the corresponding authors.

## Ethics Statement

Ethical review and approval was not required for the study on human participants in accordance with the local legislation and institutional requirements. The patients/participants provided their written informed consent to participate in this study. Written informed consent was obtained from the individual(s) for the publication of any potentially identifiable images or data included in this article.

## Author Contributions

HR and LQ designed the study. HR downloaded and analyzed the data, performed experiments, and drafted the manuscript. HR, SZ, LZ, FZ, and LQ revised the manuscript. All the authors read and approved the final manuscript.

## Funding

This work was partly supported by the National Natural Science Foundation of China (nos. 61976110, 62176112, and 11931008), Natural Science Foundation of Shandong Province (no. ZR202102270451), and the Open Project of Liaocheng University Animal Husbandry Discipline (no. 319312101-01).

## Conflict of Interest

The authors declare that the research was conducted in the absence of any commercial or financial relationships that could be construed as a potential conflictof interest.

## Publisher's Note

All claims expressed in this article are solely those of the authors and do not necessarily represent those of their affiliated organizations, or those of the publisher, the editors and the reviewers. Any product that may be evaluated in this article, or claim that may be made by its manufacturer, is not guaranteed or endorsed by the publisher.
